# Accuracy of Response Assessment FDG PET-CT Post (Chemo)Radiotherapy in HPV Negative Oropharynx Squamous Cell Carcinoma

**DOI:** 10.3390/cancers14194680

**Published:** 2022-09-26

**Authors:** Zsuzsanna Iyizoba-Ebozue, Sarah Billingsley, Russell Frood, Sriram Vaidyanathan, Andrew Scarsbrook, Robin J. D. Prestwich

**Affiliations:** 1 Department of Clinical Oncology, Leeds Cancer Centre, Leeds LS9 7TF, UK; 2 Department of Radiology, Leeds Cancer Centre, Leeds LS9 7TF, UK; 3 Leeds Institute of Health Research, University of Leeds, Leeds LS2 9NL, UK

**Keywords:** human papilloma virus, PET-CT, oropharyngeal squamous cell cancer, radiotherapy

## Abstract

**Simple Summary:**

Oropharyngeal squamous cell carcinoma is often treated with (chemo)radiotherapy with curative intent. Human papilloma virus (HPV) is a key risk factor for the development of a majority of oropharynx cancers in many parts of the world. PET-CT is widely used as an accurate method of assessing response following (chemo)radiotherapy and most of the data supporting this is based upon HPV-related disease. Oropharynx squamous cell carcinoma that is not related to human papilloma virus has an inferior prognosis and there is little data regarding the accuracy of response assessment PET-CT after chemoradiotherapy. This study shows that a negative PET-CT after treatment for patients with HPV-negative oropharynx cancer has a high negative predictive value with treatment having been successsful; however if the PET-CT is equivocal there is a significant chance of disease being persistent.

**Abstract:**

Background: Data on the accuracy of response assessment 2-[fluorine-18]-fluoro-2-deoxy-D-glucose (FDG) positron emission tomography-computed tomography (PET-CT) following (chemo)radiotherapy in patients with oropharynx squamous cell carcinoma (OPSCC) is predominantly based on HPV-positive disease. There is a paucity of data for HPV-negative disease, which has a less favourable prognosis. Methods: 96 patients treated with (chemo)radiotherapy for HPV-negative OPSCC with baseline and response assessment FDG PET-CT between 2013–2020, were analysed. PET-CT response was classified as negative, equivocal, or positive based on qualitative reporting. PET-CT response categories were analysed with reference to clinicopathological outcomes. Test characteristics were evaluated, comparing negative results to equivocal and positive results together. Post-test probabilities were calculated separately for positive and equivocal or negative results. Results: Median follow-up was 26 months. The negative predictive value of a negative scan was 93.7 and 93.2%, respectively, for primary tumour and nodal disease. For a negative scan, the post-test probability was 0.06 for primary and 0.07 for nodal disease. The post-test probability of an equivocal scan was 0.51 and 0.72 for primary and lymph node, respectively. The post-test probability of a positive scan approached 1. For patients with/without a negative scan, two-year overall survival and progression-free survival were 83% versus 30% and 79% versus 17% (*p* < 0.001), respectively. Conclusion: The NPV of a negative response assessment PET-CT in HPV-negative OPSCC is high, supporting a strategy of clinical monitoring. Contrasting with the published literature for HPV-positive OPSCC, an equivocal response scan was associated with a moderate rate of residual disease.

## 1. Introduction

Human papilloma virus (HPV) infection has been identified as a key risk factor which has driven an increase in the incidence of oropharynx squamous cell carcinoma (OPSCC) [1]. HPV-positive OPSCC is associated with a more favourable prognosis, as reflected in the recent 8th Edition of the American Joint Committee on Cancer (AJCC) staging system, which defined HPV-positive and HPV-negative OPSCC as different entities [2]. Immunohistochemistry for p16 is widely used as a reliable surrogate for HPV status [3]. Definitive (chemo)radiotherapy is a standard of care for the management of both HPV-positive and HPV-negative locally advanced OPSCC [4,5,6].

Accurate response assessment is critical following non-surgical treatment with (chemo)radiotherapy. The PET-NECK trial demonstrated that in patients in whom a complete response is achieved with 2-[Fluorine-18]-fluoro-2-deoxy-D-glucose (FDG) positron emission tomography-computed tomography (PET-CT), neck dissections can be omitted without any detriment to survival [7]. The role of PET-CT for response assessment is now integrated within UK national guidelines [4,8,9]. Response assessment PET-CT has a high negative predictive value (NPV) at the primary site and lymph nodes [10,11,12,13], facilitating the identification of patients who do not require surgical intervention. A meta-analysis reported a negative predictive value (NPV) of 95–97% for FDG PET-CT response assessment of locoregional disease, although the positive predictive value (PPV) was more limited at 64–69% [14].

HPV-positive OPSCC accounts for the majority of OPSCC in many areas of the world [15]. HPV-related OPSCC dominates the literature examining the role of FDG PET-CT in response assessment. For example, in the PET-NECK trial, 79% (*n* = 449) of patients were tested for p16 expression, with 75% (*n* = 335) being positive [7]. Other institutional series have included high proportions of HPV-positive OPSCC [16,17,18], and some have focused entirely on HPV-related disease [10,19,20]. Despite HPV-positive and HPV-negative OPSCC having very different prognoses, there is a paucity of data on the accuracy of PET-CT as a response assessment tool to guide management in HPV-negative OPSCC. There is a need to determine the predictive value of response assessment PET-CT in HPV-negative OPSCC in order to inform clinicians as to whether the PET-CT surveillance strategies used in HPV-positive disease are appropriate in HPV-negative OPSCC. This study aims to evaluate the accuracy of PET-CT following (chemo)radiotherapy in an exclusively HPV-negative OPSCC cohort.

## 2. Materials and Methods

This was a retrospective analysis of patients with HPV-negative oropharynx SCC treated between 2013 and 2020 with definitive (chemo)radiotherapy in a single institution. All patients underwent baseline and response assessment FDG PET-CT, using a negative p16 assay as a reliable surrogate for HPV negativity [3]. Formal ethics approval was waived in this study as this was considered to represent evaluation of a routine clinical service by the institutional review board. Patients were identified from an institutional database and electronic notes were reviewed to determine patients who fulfilled the eligibility criteria for analysis. Inclusion criteria were: (i) histologically confirmed OPSCC; (ii) p16 negative; (iii) definitive (chemo)radiotherapy with curative intent; (iv) FDG PET-CT as a baseline prior to treatment; and (v) FDG PET-CT as response assessment. Scoring of p16 immunohistochemistry was performed using a threshold for strong and diffuse nuclear and cytoplasmic staining of ≥70% in the tumour [21]. Exclusion criteria were (i) history of prior therapeutic resection of primary and/or lymph node disease and (ii) PET-CT only done at baseline or only used to assess response after detection of abnormalities on CT and/or MRI.

Demographic, clinical, and imaging data, including PET-CT findings and maximum standardised uptake value (SUVmax) at the primary site and lymph nodes (at presentation and at response assessment), were obtained from a review of electronic case note records. Staging information was derived from physical examination, nasoendoscopy, examination under anaesthetic with biopsy where indicated, MRI or contrast-enhanced CT of the head and neck region, and CT of the thorax. In routine practice PET-CT is performed on clinician discretion, and is generally used for staging and as a baseline for future response assessment patients with stage II or stage III/IV disease. Results of PET-CT were routinely reviewed in a specialist head and neck multidisciplinary meeting. For the purposes of this study, AJCC TNM staging (8th Edition) [2] was assigned utilising all available clinical and radiological information (part of the cohort of patients had been originally staged as part of routine care under the TNM 7 classification).

### 2.1. Radiotherapy

Radiotherapy techniques and approach to target delineation changed over the study period. In the early part of this retrospective cohort, target delineation was performed using a compartmental approach, with this outline being changed to a volumetric approach during this period. By 2016, the clinical target volume (CTV) was created via geometric expansion of the gross tumour volume by 10 mm (modified to anatomical boundaries). In 2018, following updated international consensus guidelines [22] for CTV delineation, a 5 + 5 mm approach was adopted in appropriate cases. The planning target volume was created by auto-expansion of the CTV by 4 mm. Institutional protocols were followed as previously described [23], including prospective peer review [24], with a radical treatment dose of 70 Gy in 35 fractions over 7 weeks or 65 Gy in 30 fractions over 6 weeks, with lower doses to prophylactic dose regions (54–57 Gy in 30–35 fractions over 6–7 weeks). Treatment delivery was carried out using the volumetric arc therapy (VMAT) technique.

### 2.2. Follow Up

Tumour response was routinely assessed 4 months after the completion of treatment by clinical examination, nasoendoscopy, and PET-CT. The results of these response assessments were routinely discussed in multi-disciplinary team (MDT) meetings; decisions as to whether to proceed with examination under anaesthesia and/or biopsies were generally carried out based on the degree of clinical concern based on examination/nasoendoscopy and/or PET-CT that there might be residual disease and whether there was considered to be a possibility of salvage surgery in the event of residual disease. For certain patients with an indeterminate response on initial response assessment PET-CT with a low clinical suspicion of residual disease a repeat or ‘second look’, PET-CT was considered following MDT discussion after an interval of approximately 3 months, following the previously reported approach [25]. Patients were routinely followed up for a total of 5 years prior to discharge.

### 2.3. PET-CT Protocol

FDG PET-CT examinations from June 2013 to October 2014 were carried out on a 64-slice Philips Gemini TF64 scanner (Philips Healthcare, Amsterdam, The Netherlands) and after October 2014 on a 64-slice Discovery 710 scanner (GE Healthcare, Chicago, IL, USA). Patients were routinely imaged on the same scanner for baseline and response assessment PET-CT, except for a small number of patients who were treated shortly before the original scanner was replaced. Serum blood glucose was routinely checked, and if >10 mmol/L, scanning was not carried out. Patients fasted for 6 h prior to intravenous fluorine-18 FDG injection; the dose varied according to patient body weight. PET acquisition from the skull vertex to the upper thighs was carried out 60 min after tracer injection. A silence protocol was used in the uptake period after tracer injection to minimise physiological tracer activity within the head and neck region. The CT component was performed according to a standardised protocol (without the use of iodinated contrast medium) with the following settings: 140 kV; 80 mAs; tube rotation time 0.5 s per rotation; pitch 6; section thickness 3.75 mm (to match the PET section thickness). Patients maintained normal shallow respiration during CT acquisition. Images were reconstructed using a standard ordered subset expectation maximisation algorithm with CT for attenuation correction. Both non-attenuation-corrected and attenuation-corrected datasets were reconstructed.

### 2.4. Categorisation of PET-CT Response

For analysis, categorisation of PET-CT response was based upon formal PET-CT reports, as previously described in [25,26]. As part of routine clinical practice, all PET-CT scans were reported by experienced radiologists dual-certified in radiology and nuclear medicine. The standard approach to reporting was for qualitative assessment of PET-CT images (by comparison of tumour or nodal tracer activity with background physiological uptake in the soft tissues and liver). While semi-quantitative assessment (SUVmax) of residual tumour or nodal uptake was documented, this was not fundamental to the qualitative interpretation of response. Primary tumour and nodal SUVmax values were documented. The results of post-treatment PET-CT were categorised as positive, equivocal, or negative for the primary site and nodal sites separately. A result was classified as positive if focal uptake was present, corresponding to a structural abnormality, and of greater intensity than background liver activity. Scans were classed as equivocal if focal FDG uptake was reduced from baseline while being below liver background and above that of surrounding normal tissues. Scans were classed as negative in the absence of any abnormal focal FDG uptake or diffuse FDG uptake in the absence of corresponding anatomical abnormality on the CT that was considered to represent radiotherapy related inflammatory activity (i.e., complete metabolic response). The presence or absence of residual tissue on the CT component of the post treatment PET-CT was recorded.

### 2.5. Analysis and Statistics

Follow-up duration was defined from the final fraction of radiotherapy. Disease status post-treatment was categorised based on review of clinical records and any radiology or pathology. For patients who did not undergo a biopsy/surgical intervention, serial negative physical examinations over the follow-up period and any relevant imaging investigations were used as confirmation of disease status and categorised into local/regional/distant disease sites.

Sensitivity, specificity, PPV, and NPV were calculated using two-by-two tables constructed using clinico-pathological outcomes. Post-test probabilities were calculated for positive, equivocal, or negative scans for primary site and lymph separately; likelihood ratios were not used to avoid zero error and likelihood ratios approaching infinity, as all patients with a positive scan had evidence of residual disease. Kaplan–Meier and log-rank tests (*p*-values with *p* < 0.05 considered significant) were performed for survival analysis.

## 3. Results

### 3.1. Cohort Characteristics

**Figure 1:**
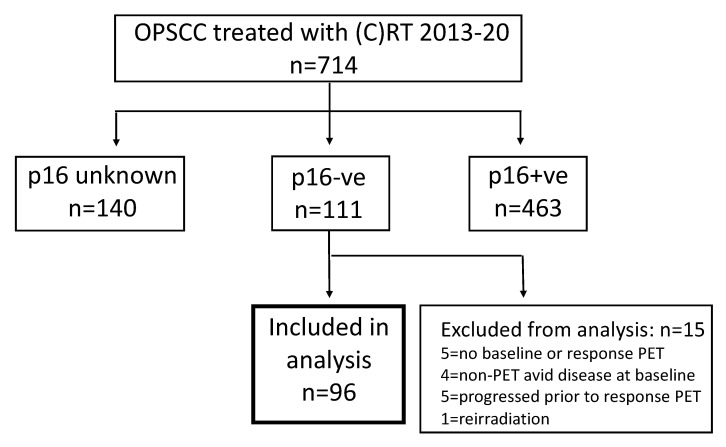
Flowchart showing identification of patients with p16 negative OPSCC included in analysis.

A total of 714 patients with OPSCC were treated with radiotherapy during the study period. Figure 1 shows the selection of patients for analysis with p16 negative OPSCC. The p16 status was unknown in 140 patients, and of the remaining patients 463 were p16 positive and 111 (24.0% of patients with known p16 status) were p16 negative. Fifteen patients with p16 negative OPSCC were excluded from analysis for the following reasons: five did not have baseline or response assessment PET-CT, five progressed prior to response assessment, four had non-avid baseline disease, and one was receiving re-irradiation. Therefore, 96 patients with p16 negative OPSCC met the inclusion criteria and formed the study cohort.

Table 1 summarises the patients’ demographic and treatment details. Concurrent platinum-based chemotherapy was delivered in 60/96 (61.5%) of patients. Reasons for not delivering chemotherapy were age > 70 (*n* = 14), limited performance status (*n* = 6), alcohol-related issues (*n* = 5), cerebrovascular disease (*n* = 4), renal comorbidity (*n* = 2), and other (*n* = 4). The median follow-up time was 26 months (range 3–60).

### 3.2. Outcomes

The two-year survival estimates for the whole cohort were 60% for overall survival (OS), 52% for progression free survival (PFS), 77% local control, 67% regional control, and 81% distant control; 52/96 (54%) patients died during the follow up period. Overall, 39/96 (40.6%) patients had disease progression based on clinical, radiological, and/or pathological findings. In those who progressed, locoregional progression occurred in 28/39 patients (72%) and primary progression was seen in 23/39 (56%) patients. Primary progression alone in the absence of regional nodal or distant progression occurred in five patients. Lymph node progression occurred in 23/39 (56%), with nodal disease occurring without progression of the primary tumour or development of distant metastases in four patients. Distant metastatic disease was observed in 16/39 (41%) patients (all based on radiological findings), with lung metastases in 11/16 of these patients; 5/16 patients also had primary tumour progression and 9/16 also had regional lymph node progression. Two patients with disease progression were successful salvaged with surgery, subsequently remaining disease free: wedge resection for solitary lung metastasis in a patient with only distant progression, and oropharyngeal resection plus ipsilateral neck dissection in a patient with only primary progression.

### 3.3. Assessment of Response by PET-CT and Correlation with Outcome

All patients had a baseline pre-treatment PET-CT. The median time to response assessment PET-CT following completion of (chemo)radiotherapy was 17 weeks (interquartile range 16–17 weeks).

Overall, on response assessment PET-CT 54/96 (56.3%) patients had a negative scan, 20/96 (20.8%) patients had an equivocal response, and 22/96 (22.9%) had positive scans with FDG-avid residual disease. In addition, 8/96 patients were found to have new distant metastases, and in each of these patients there was residual locoregional disease on PET-CT.

**Figure 2:**
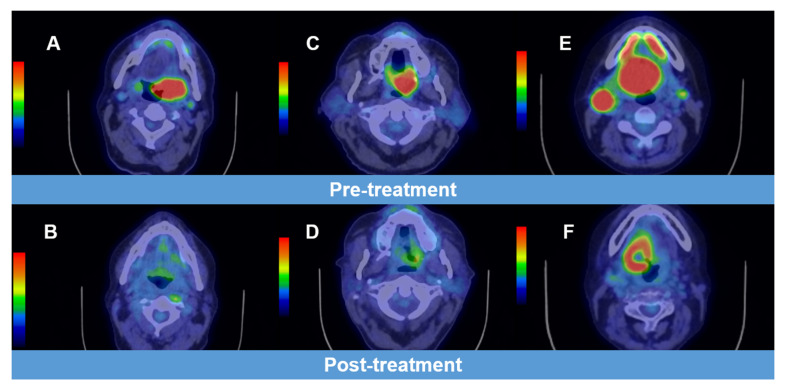
Examples of imaging illustrating positive, equivocal, and negative results on response assessment PET-CT. (**A**) Baseline PET-CT for a patient with T4N1M0 p16-ve squamous cell carcinoma of the tonsil (SUVmax 23.5, with (**B**) showing a negative response assessment PET-CT (absent abnormal focal FDG uptake). (**C**) Baseline PET-CT for a patient with T4N2bM0 p16-ve squamous cell carcinoma of the soft palate (SUVmax 15.3), with (**D**) showing an equivocal response on assessment PET-CT (SUVmax 4.2). (**E**) Baseline PET-CT for a patient with SCC T4N2cM0 p16-ve squamous cell carcinoma of the base of the tongue (SUVmax 13.4), with (**F**) showing a positive response on assessment PET-CT (SUVmax 10.5).

On baseline PET-CT scans, the primary tumour was FDG avid in 93/96 patients, with a mean SUVmax of 14 (range 4.2–33.1). The primary was non-FDG avid in three patients and biopsy proven; the primary tumour response in these patients was not considered assessable by PET-CT. In the primary tumour on response assessment PET-CT, 64/93 (68.8%) patients had a negative scan, 21/93 (22.5%) had an equivocal response, and FDG-positive residual disease was detected in 8/93 (8.6%) (see Figure 2).

Regarding nodal disease, 81/96 patients had lymph node disease based on pre-treatment cross-sectional imaging (CT and/or MRI), and PET-CT. 77/81 (95%) of these patients had FDG-avid lymph node disease at baseline with a mean SUVmax of 8.9 (range 2.5 to 23.6). Meanwhile, 4/81 (4.9%) had non-avid nodal disease (abnormal lymph nodes on MRI in all four patients, cytological confirmation in one). On response assessment PET-CT, 59/77 (76.6%) had a negative scan. Equivocal and residual FDG-positive nodes were reported in 11/77 (14.2%) and 7/77 (9%) patients, respectively.

Table 2 summarises the accuracy of PET-CT, comparing complete versus non-complete responses (equivocal and positive scans grouped together for analysis) for the primary site (*n* = 93 assessable by PET), lymph nodes (*n* = 77 assessable by PET), and overall (*n* = 96).

Post-test probabilities for PET-CT response primary site and lymph node outcomes, respectively, were analysed as three separate response categories: negative, equivocal, and positive. These are summarised in Table 3 and Table 4, respectively. For a negative scan, the post-test probability was 0.06 for primary and 0.07 for nodal disease. The post-test probability of an equivocal scan was 0.51 and 0.72 for primary and lymph nodes, respectively. The post-test probability of a positive scan approached 1.

**Figure 3:**
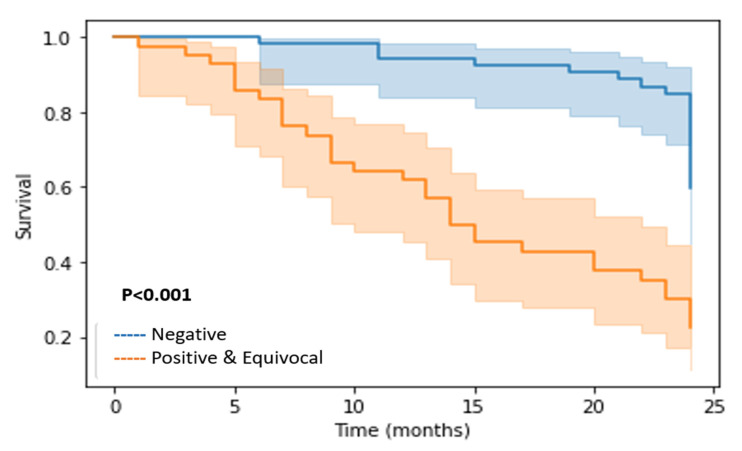
Kaplan–Meier (K-M) curve for two-year survival (OS). K-M curve for two-year OS comparing negative scans versus equivocal and positive scans grouped together.

There were significant differences between all the estimated two-year outcomes between patients with and without a negative scan (all being *p* < 0.001), with OS being 83% versus 30%, PFS 79% versus 17%, local control 96% versus 52%, regional control 92% versus 33%, and distant control 94% versus 65%. The Kaplan–Meier curves for two-year OS are depicted in Figure 3.

## 4. Discussion

Surveillance strategies following (chemo)radiotherapy for OPSCC rely on the accuracy of response assessment FDG PET-CT for both primary and nodal disease. A high NPV is key to safely avoiding neck dissection and unnecessary investigations/biopsies of the primary site [7,11,12]. A recent meta-analysis reported an NPV of 95–97% [14]. PPV varies considerably between series, and partly depends on the approach taken to classification of response scans; for example, ‘equivocal’ scans can be analysed together or separately from ‘positive’ scans [26]. In the meta-analysis, PPV was reported as 64–69% [14]. A recent series assessing FDG PET-CT performed 12 weeks post-treatment reported that in patients with HPV-related OPSCC the PPV was low, at 30%, with OS of ‘equivocal’ responders in HPV-positive patients being no different than complete responders [16]. It has been recognised that HPV-positive and HPV-negative OPSCC have differing rates of anatomical response on cross-sectional imaging, with HPV-related disease demonstrating greater regression by 12 weeks post-treatment but taking longer to completely regress than HPV-negative disease (with ongoing shrinkage reported up to 36 weeks) [27]. The limited PPV of FDG PET-CT, along with recognition of the extended time needed for HPV-related OPSCC to reach maximum radiological response, has led to the development of a strategy of a ‘second look’ PET-CT for certain patients with equivocal findings on initial post-treatment PET-CT [16,25,28].

Reported PET-CT series generally have high proportions of HPV-related OPSCC. HPV-negative OPSCC has a poorer prognosis [1] and would be expected to have a higher prevalence of residual disease at the point of response assessment. This difference may be critical to the value of PET-CT in guiding a surveillance strategy based on high NPV and the potential use of surveillance following an equivocal response.

The current analysis of the accuracy of response assessment PET-CT is, to the best of our knowledge, the largest reported in an HPV-negative OPSCC cohort. The overall outcomes of patients are limited, with a two-year OS of 60%, which is in line with the expected poorer prognosis of HPV-negative disease. Despite the moderate prevalence of residual disease post-treatment, the NPV remained high for both primary (93.7%) and lymph node disease (93.2%). Analysing positive and equivocal scans together, the PPVs for the primary site and lymph nodes were 65.5% and 83.3%, respectively. Analysis of positive and equivocal responses separately showed that the post-test probability of a positive scan approached 1, while the probability of an equivocal scan was 0.51 and 0.72 for primary and lymph node, respectively.

Urban et al. [18] performed a retrospective review to examine the performance of response assessment PET-CT following (chemo)radiotherapy for oropharynx cancer patients stratified by p16 status; this study included 92 patients with p16 negative disease. This study reported a very high NPV of at least 99%, regardless of p16 status, for both primary site and regional lymph nodes. The primary PPV was 26% for p16 positive and 54% for p16 negative; similarly, the regional PPV was 31% for p16 positive and 58% for p16 negative. Other series have included smaller numbers of HPV-negative OPSCC patients. Awan et al. [29] analysed 68 patients with p16 positive and 40 patients with p16 negative head and neck squamous cell carcinoma; primary disease the PPV for was 50%, while it was 72.7% for p16 positive and negative; regional recurrence was 33% versus 66.6%, respectively. The NPV for local recurrence was 100%, while for regional recurrence it was 100% for p16 positive patients and 88.5% for p16 negative patients. Rulach et al. [16] reported a series of 135 HPV positive and 20 HPV negative patients; the PPV of 12-week response assessment PET-CT was 30% and 81.8% in HPV-positive and HPV-negative patients, respectively, while the NPVs were 92.9% and 55.6%, respectively.

Overall, the data from the two largest series (the current series and Urban et al. [18]) suggest that a negative scan has a high NPV and could safely be used to avoid surgical intervention, as is standard practice for HPV-positive OPSCC. In a series of predominantly HPV-positive OPSCC, we have previously reported a low PPV (20%) for equivocal responses in lymph nodes [12]; this is the basis of the strategy of a ‘second look’ PET-CT to avoid unnecessary surgical intervention in the context of a low probability of residual disease [25,28]. However, these series in HPV-negative OPSCC patients suggest that, in contrast to HPV-positive OPSCC, the PPV of HPV-negative disease is considerably higher and equivocal responses have a high probability of harbouring residual disease. Therefore, the approaches to ongoing surveillance with repeat PET-CT advocated for HPV-positive OPSCC following an equivocal response with a low PPV [16,25,28] do not appear suitable for HPV-negative disease due to its much higher probability of residual disease and the risk that delay will reduce the possibility of successful salvage surgery.

It is interesting to note that these findings regarding the accuracy of response assessment PET-CT are similar to those we have previously reported in patients treated with radiotherapy alone (no chemotherapy), who would be expected to have a higher prevalence of residual disease [26]. In that series, the NPV of PET-CT remained high (91% for lymph node disease), while the PPV was also high (83% for lymph node disease).

The strengths of this study include a focus on an HPV-negative OPSCC cohort, a clearly defined imaging strategy with baseline and response PET-CT, and the use of definitions of positive, equivocal, and negative scans which reflect ongoing real-world clinical practice. Our approach throughout this time period has been to aim to perform response assessment PET-CT at a 4-month post-treatment timepoint. The rationale for this is the knowledge of protracted involution post-treatment [27] and the superior accuracy of PET-CT at least 12 weeks post-treatment compared with earlier PET-CT.

There are several limitations to the study. Although 96 patients represents a large series in the context of HPV-negative disease, the sample size remains limited. In the initial part of the study period, p16 testing was not used for all patients, meaning that certain patients might have had unknown p16 status. Reflective of clinical practice, the series is heterogenous in terms of disease stage and treatment, with 36% of patients treated with radiotherapy alone and variations in the timing of response assessment PET-CT. Follow up duration was limited (median 26 months). The qualitative nature of PET-CT reporting may cause issues with reproducibility in other centres. However, there is no consensus regarding optimal interpretive criteria; we have previously compared multiple interpretive criteria in a large cohort and found that the methods used here in clinical practice were among those providing an optimal balance between minimising equivocal findings and maintaining a high NPV [17].

## 5. Conclusions

The accuracy of FDG PET-CT for OPSCC response assessment is partly dependent on whether the disease is p16 positive or negative. The NPV of a negative assessment by PET-CT in HPV-negative OPSCC is high, supporting the strategy of observation widely used in the management of HPV-positive OPSCC. In contrast with the published literature for HPV-positive OPSCC, an equivocal response scan was associated with a moderate rate of residual disease, suggesting that repeat imaging/surveillance may not be an appropriate strategy.

## Figures and Tables

**Table 1:** Patient demographics.

	*n* = 96	%
Age (Median, Years)	60	
Sex		
• Male	71	74%
• Female	25	26%
Tumour subsite		
• Tonsil	50	27.1%
• Base of tongue	26	52.1%
• Vallecula	3	3.1%
• Posterior pharyngeal wall	7	7.3%
• Soft palate	10	10.4%
T stage		
• T1	8	8.3%
• T2	36	37.5%
• T3	29	30.2%
• T4	23	24%
N stage		
• N0	15	15.6%
• N1	15	14.9%
• N2a	2	2.1%
• N2b	42	43.75%
• N2c	22	22.9%
• N3	0	0%
Staging		
• I	1	1%
• II	6	6.3%
• III	17	17.7%
• IV	72	75%
Smoking		
• Current Smoker	55	57.3%
• Ex-smoker	31	32.3%
• Never smoked	10	10.4%
Histopathology/Grade		
• 1	3	3.1%
• 2	39	40.6%
• 3	45	46.9%
• Unknown	9	9.4%
Fractionation		
• 70 Gy/35#	84	87.5%
• 65 Gy/30#	12	12.5%
Radiotherapy alone	35	36.4%
Concurrent chemotherapy	61	63.5%
Agent		
• Cisplatin	44	72.1%
• Carbo	8	13.1%
• Cis/Carbo	8	13.1%
• Cetuximab	1	1.6%
No of cycles		
• 1	2	3.33%
• 2	35	58.33%
• 3	20	33.33%
• >3	3	5
Induction chemotherapy		
• TPF	2	3.3%

**Table 2:** Details of accuracy of PET-CT response scans comparing complete metabolic response versus non-complete responses.

	Primary Site *n* = 93	Neck Nodes *n* = 77	Overall Primary, Neck Nodes and Distant *n* = 96
PET-CT positive (equivocal and positive)	29	18	42
PET-CT negative	64	59	54
True positive	19	15	31
True negative	60	55	46
False positive	10	3	11
False negative	4	4	8
Sensitivity	82.6%	78.9%	79.4%
Specificity	85.7%	94.8%	75.4%
Positive predictive value	65.5%	83.3%	73.8%
Negative predictive value	93.7%	93.2%	85.2%
Accuracy	84.9%	90.9%	80.2%

**Table 3:** Primary tumour response (*n* = 93).

PET-CT Response Scan Outcome for FDG-Avid Primary Site	Number	Disease Status on Follow-UpDisease +	Disease Status on Follow-UpDisease −	Post-Test Probability
Positive	8	8	0	0.99
Negative	64	4	60	0.06
Equivocal	21	11	10	0.51
Total	93	23	70	

**Table 4:** Lymph node response (*n* = 77).

PET-CT Response Scan Outcome for FDG-Avid Nodal Disease	Number	Disease Status on Follow-Up Disease +	Disease Status on Follow-UpDisease −	Post-Test Probability
Positive	7	7	0	0.99
Negative	59	4	55	0.07
Equivocal	11	8	3	0.72
Total	77	19	58	

## Data Availability

The data presented in this study are available on request from the corresponding author.
